# Antiepileptic drug exposure in pregnancy and pregnancy outcome from national drug usage data

**DOI:** 10.1186/s12884-018-1728-y

**Published:** 2018-04-06

**Authors:** Noni Richards, David Reith, Michael Stitely, Alesha Smith

**Affiliations:** 1 School of Pharmacy, University of Otago, 18 Frederick St, Dunedin, New Zealand; 2 Department of Women’s and Children’s Health, Dunedin School of Medicine, University of Otago, Dunedin, New Zealand; 3 bpacnz, 10 George St, Dunedin, New Zealand

**Keywords:** Antiepileptics, Pregnancy, Spontaneous abortion, Termination, Induced abortion, Administrative databases, Epilepsy, Bipolar disorder

## Abstract

**Background:**

Antiepileptic drugs (AEDs) are used by pregnant women to manage conditions such as epilepsy and bipolar disorder even though they pose a risk to the developing foetus. This study aimed to determine the overall use of AEDs by women during their childbearing years and women who are pregnant and the association between AED use and rates of pregnancy termination and spontaneous abortion.

**Methods:**

Retrospective population based cohort study using administrative databases in New Zealand between 2008 and 2014. Women who had been pregnant were identified by the National Minimum Dataset and were linked to the Pharmaceutical Collection to obtain information on use of AEDs. Women aged between 15 and 45 years dispensed AEDs were identified in the Pharmaceutical Collection.

**Results:**

There was an increase in the number of women of child-bearing potential prescribed AEDs, from 9 women per 1000 women in 2008 to 11.4 women per 1000 women in 2014. Women who had been dispensed an AED had an increased rate of spontaneous abortion 8.97 spontaneous abortions per 100 pregnancies, compared with, 6.31 per 100 pregnancies (risk ratio 1.42, 95% CI 1.40 to 1.44), and a decreased rate of pregnancy termination, 18.51 terminations per 100 pregnancies compared with 19.58 per 100 pregnancies (risk ratio 1.95, 95% CI 0.94–0.96).

**Conclusion:**

Use of newer AEDs is increasing in women of child-bearing potential in New Zealand leading to an overall increase in AED use in this group despite a fall in the use of older AEDs. AED use is this study was associated with an increased risk of spontaneous abortion and decreased rate of pregnancy termination, however confounding by indication could not be excluded.

## Key findings from study

- The number of women of child bearing potential who are taking an AED is increasing over time with General Practitioners being the most common prescribers of AEDs to this group.
- The rate of spontaneous abortion is higher for women who have been dispensed an AED.

## Background

Antiepileptic drugs (AEDs) are used by pregnant women even though they pose a risk to the developing foetus. They increase the risk of congenital malformations [1] and some, particularly sodium valproate, can cause developmental delay in children when used during pregnancy [2–4]. AED use during pregnancy has also been associated with other potential obstetric risks such as postpartum haemorrhage and intrauterine growth restriction, [5, 6] and some research suggests they increase the risk of spontaneous abortion and stillbirth although evidence is conflicting [6, 7]. Despite these risks, women with epilepsy typically continue treatment with AEDs during pregnancy to avoid the potentially harmful effect of recurrent seizures which can have significant long term neurological and physical consequences to both themselves and their foetuses [8, 9]. Likewise, women with bipolar disorder often continue treatment with AEDs during pregnancy to minimise the risk of mood episode recurrence and its associated morbidity [10]. AEDs are also utilised for other neurological conditions such as migraine and pain.

Internationally researchers have found that overall use of AEDs is increasing despite declining use of the older generation AEDs [11]. This is thought to be the result of increasing use of the newer AEDs such as lamotrigine and levetiracetam and the utilisation of AEDs for a wider range of neurological uses, in particular for psychiatric conditions such as bipolar disorder [12, 13]. As a result there can be a wide range of health professionals who prescribe AEDs and this can create issues with continuity of care, particularly concerning the prescribing of concomitant birth control or providing preconception counselling in women of child-bearing age [14].

The aim of this whole of population study was to investigate the use of AEDs by women in New Zealand and determine pregnancy outcomes for women dispensed AEDs during pregnancy. The specific objectives were to determine the overall use of AEDs and patterns of use by women during their childbearing years and women who are pregnant in New Zealand and the association between AED use and rates of pregnancy termination and spontaneous abortion.

## Methods

### Study population

Using the National Health Index (NHI) all records from the Pharmaceutical Collection were linked to the National Minimum Dataset (NMDS) to identify women with no pregnancy, a birth, spontaneous abortion or pregnancy termination, to create the following cohorts:

1. Women of childbearing age (15–45 years old)
  i) women aged between 15 and 45 years old who were dispensed a study AED three or more times in a 12 month period between January 1, 2008 and December 31, 2014.
  ii) estimated population of women in New Zealand aged between 15 and 45 years old [15].
2. Pregnant women
  - women who had a spontaneous abortion or pregnancy termination between January 1, 2009 and December 31, 2014 who:
    i) had been dispensed any AED included in the study in the preceding 3 months.
    ii) had NOT been dispensed any AED included in the study in the preceding 3 months.
  - women in New Zealand who had a birth (including stillbirth) between January 1, 2009 and December 31, 2014 who:
    i) had been dispensed any AED included in the study in the preceding 9 months.
    ii) had NOT been dispensed any AED included in the study in the preceding 9 months.

Every person in New Zealand has a unique NHI number, an alphanumeric identifier that is used in all interactions with the health system over their life. This number makes it possible to link an individual’s health data across a range of databases. The recording of NHIs are reliable from 2008, with 97% of all records containing an NHI therefore this study used data from the NMDS and the Pharmaceutical Collection between 2008 and 2014. Pregnancy outcomes starting from January 2009 were used to ensure consistent NHI recording 9 months prior.

The NMDS is a national collection of public and private hospital discharge information, including coded clinical data for both inpatients and day patients. Every hospital birth event (approximately 97% of births in New Zealand) is captured by the NMDS. The NMDS also contains information about pregnancy terminations and spontaneous abortions that are managed in public hospitals. The Pharmaceutical Collection contains claim and payment information from pharmacists for all subsidised community dispensing in New Zealand (100% of all subsidised medicines), however not those dispensed in hospital.

All study data contained only encrypted NHI numbers and no identifying details such as name or address were used. Any women missing an NHI number or with more than 20% of their data missing were excluded.

### Pregnancy outcomes

To capture pregnancy outcomes in the NMDS database ICD 10 codes for abortion, ectopic pregnancy and birth were used, grouped as follows: spontaneous abortions (O02 – O039), pregnancy terminations or induced abortions (O040 – O049), and births, including stillbirths (Z370 – Z375). Molar (O01) and ectopic pregnancies (O00) were excluded. To help ensure validity of the data and minimise incorrect coding issues, women with maternity codes who were under 15 years old or over 55 years old were excluded. Each pregnancy in the study time period was included.

Abortions are defined in New Zealand as foetal loss usually during the first 20 weeks of gestation. Induced abortions (pregnancy terminations) are those initiated voluntarily with the intent of terminating a pregnancy. All other abortions are considered spontaneous abortions. The legal definition of stillbirth in New Zealand is a child born dead who weighs 400 g or more or who is born after 20 completed weeks gestation.

### Assessment of AED exposure

In New Zealand, all AEDs are supplied by prescription and are subsidised if listed in the pharmaceutical schedule. The use of AEDs was defined as any prescription redeemed with drugs from the ATC code group Antiepileptics (N03AA) or subsidised under the “control of epilepsy” section of the schedule. This included the following medicines: carbamazepine, clobazam, clonazepam, ethosuximide, gabapentin, lacosamide, lamotrigine, levetiracetam, primidone, sodium valproate, topiramate and vigabatrin [16]. Dispensing information about the use of AEDs which are not subsidised, such as pregabalin and oxcarbazepine, is not captured by the pharmaceutical collection.

We defined any dispensing of an AED within 9 months of a birth or stillbirth or within 3 months of a spontaneous abortion or termination as an exposure to an AED in pregnancy. The date the medicine was dispensed determined at which stage during the pregnancy the exposure to an AED occurred. Exposure to polypharmacy was recorded for women who were dispensed two or more AEDs in the same trimester.

### Statistical analysis

Rates of use (per population) and trends over time were determined using SPSS for pregnant women who were dispensed AEDs during the exposure period and for women of child bearing age (15–45 years) dispensed AEDs. Analysis of individual AEDs involved comparison of column proportions using z tests with a significance level of 0.05 (adjusted using the Bonferroni correction).

Incidence rates and risk ratios were generated for pregnancy outcomes comparing the cohort of women exposed to AED during pregnancy to those not exposed to AEDs during pregnancy. Advanced maternal age is a known risk factor for spontaneous abortion therefore risk ratios for spontaneous abortion and induced abortions were adjusted for maternal age by direct age-standardisation. Statistical significance was assumed if *p* < 0.05.

### Ethical approval

Approval for this study was granted by Human Research Ethics Committee at the University of Otago (HD15/020).

## Results

### Women of child-bearing potential

**Fig. 1:**
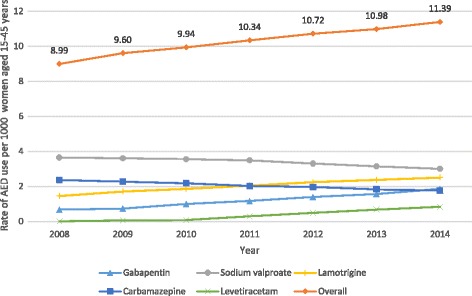
Number of women of child bearing age (15–45 years) dispensed an AED 2008–2014

**Fig. 2:**
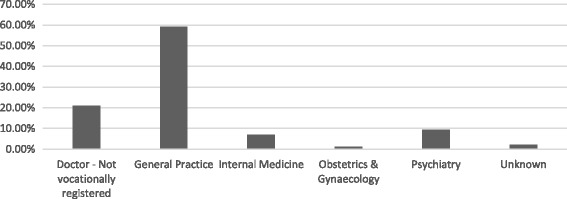
AED prescribing to women of child-bearing age by provider type 2008–2014

Over the study period, the number of women of childbearing age (between 15 and 45 years) dispensed any AED increased, from 9 women per 1000 women in 2008 to 11.4 women per 1000 women in 2014 (Fig. 1). During this time period, numbers of women dispensed sodium valproate or carbamazepine declined while the numbers of women dispensed lamotrigine, gabapentin, and levetiracetam increased. Prescriptions of AEDs to women of child-bearing age are most commonly provided by general practitioners, accounting for 60% of the prescriptions from 2008 to 2014 (Fig. 2).

### Pregnant women

2284 individual women received an AED in the 9 months before a birth, or 3 months before a pregnancy termination or spontaneous abortion between 2009 and 2014. 311 women had two pregnancies over the study period and 59 women had three or more pregnancies over this period which resulted in 2728 pregnancies. Table 1 shows the maternal age characteristics. Women dispensed an AED during this period were more likely to be older than women who had not been dispensed an AED (*p* < 0.05).

**Table 1:** Pregnancies by maternal age (years) 2009–2014

Using AEDs (*n* = 2943)					Not using AEDs (*n* = 469,816)			
Age band (years)	*Spontaneous abortion*	*Pregnancy termination*	*Birth*	Total	*Spontaneous abortion*	*Pregnancy termination*	*Birth*	Total
15–19	8	26	66	110 (3.7)	1461	16,427	17,773	35,661 (7.6)
20–24	33	112	349	494 (18.1)	4175	28,612	62,266	95,053 (20.2)
25–29	47	130	520	697 (25.5)	5652	19,779	85,962	111,393 (23.7)
30–34	65	105	547	717 (26.3)	7371	13,430	99,448	120,249 (25.6)
35–39	56	72	346	474 (17.4)	6746	9275	64,947	80,968 (17.2)
40–44	42	45	140	227 (8.3)	3734	3772	17,176	24,682 (5.3)
> 45	4	6	9	19 (0.7)	485	316	1009	1810 (0.4)
Total	255	496	1977	2728 (100.0)	29,624	91,611	348,581	469,816 (100.0)

Overall, women who had been dispensed an AED in the exposure period (within 9 months of a birth or stillbirth or within 3 months of a spontaneous abortion or pregnancy termination) had an increased rate of spontaneous abortion, 8.97 per 100 pregnancies, than those not dispensed an AED, 6.31 per 100 pregnancies (age-adjusted risk ratio 1.42, 95% CI 1.40 to 1.44) (Table 2). Women dispensed an AED had a decreased rate of pregnancy termination, 18.51 per 100 pregnancies compared with 19.50 per 100 pregnancies for women who had not been dispensed an AED (age-adjusted risk ratio 0.95, 95% CI 0.94 – 0.96).

**Table 2:** Association between AED use during pregnancy and spontaneous abortion and pregnancy termination rates

Spontaneous abortion				
AED use	Spontaneous abortion	Total Pregnancies	Incidence rate^a^ (95% CI)	Risk ratio (95% CI)
AEDs	255	2728	9.35 (8.31 to 10.50)	1.48 (1.32–1.67) *P* < 0.0001
			Age-adjusted rate^b^ 8.97 (8.89 to 9.05)	Age adjusted risk ratio^b^ 1.42 (1.40–1.44) *P* < 0.0001
No AEDs	29,624	469,816	6.31 (6.24 to 6.38)	
Pregnancy termination				
AED use	Pregnancy termination	Total Pregnancies	Incidence rate^a^ (95% CI)	Risk ratio (95% CI)
AEDs	496	2728	18.18 (16.78 to 19.67)	0.93 (0.86–1.01) *P* = 0.0860
			Age-adjusted rate^b^ 18.51 (18.40 to 18.62)	Age adjusted risk ratio^b^ 0.95 (0.94–0.96) *P* < 0.0001
No AEDs	91,998	469,816	19.58 (19.47 to 19.70)	

^a^Per 100 pregnancies; ^b^ Age-adjusted rate calculated using the cohort of unexposed women as a reference group

Analysis of pregnancy outcomes by AED, where women were only exposed to one AED during pregnancy and where there were 50 or more exposures, revealed no difference in the rate of spontaneous abortions between AEDs. Women that had a termination were more likely to be taking clonazepam, gabapentin or sodium valproate than lamotrigine or carbamazepine. Women that gave birth were more likely to be taking carbamazepine or lamotrigine than clonazepam, gabapentin or sodium valproate.

**Fig. 3:**
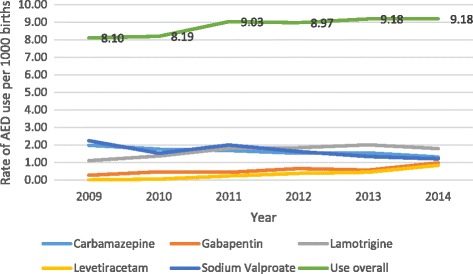
Rate of AED use by pregnant women (per 1000 births)

Overall use of AEDs by pregnant women increased slightly from 8.10 per 1000 births in 2009 to 9.18 per 1000 births in 2014. During this time, use of sodium valproate by pregnant women nearly halved from 2.24 women per 1000 births to 1.20 women per 1000 births while use of gabapentin (0.27 to 0.97 women per 1000 births), lamotrigine (1.10 to 1.79 women per 1000 births) and levetiracetam (from 0 to 0.83 women per 1000 births) increased among pregnant women (Fig. 3).

The majority of pregnant women dispensed AEDs during pregnancy are on monotherapy, only a small proportion (10.7%) were on AED polytherapy during pregnancy. In the time period from 2009 to 2014, of the women dispensed any AED during the nine months before birth, 171 (8.65%) were dispensed two different AEDs in the same trimester. 35 women (1.77%) were dispensed three AEDs and five (0.25%) women were dispensed four AEDs in the nine months before birth.

## Discussion

In this population based cohort study, we observed a higher rate of spontaneous abortion and lower rate of pregnancy termination in women who had been dispensed AEDs compared with women who had not been dispensed AEDs during pregnancy. There was also a statistically significant increase in the number of women of child-bearing age being dispensed AEDs over the study time period. Use of the sodium valproate declined over the time period while use of the newer generation AEDs, such as lamotrigine, increased. A similar trend was seen among women who were pregnant however this did not reach statistical significance. The majority of pregnant women dispensed AEDs during pregnancy are on monotherapy. With most prescriptions for AEDs in women of child-bearing potential provided by general practitioners.

It is inherently difficult to investigate the rate of spontaneous abortion in women treated with AEDs because they often occur early in pregnancy and may not be recognised and recorded. However studies which have investigated pregnancy outcomes, including rates of spontaneous abortion, after exposure to AEDs during pregnancy have found conflicting results [6, 17]. A recent large meta-analysis assessing the association between epilepsy, AED exposure and pregnancy outcomes found that women with a diagnosis of epilepsy had a significantly increased rate of spontaneous abortion however exposure to AEDs was not associated with an increased risk [6]. Another study found a small but statistically significant elevated risk of spontaneous abortion in women who took AEDs but concluded that it was likely to be the result of confounding by indication [17]. This present study found an increased rate of spontaneous abortion among women prescribed AEDs. While these results were statistically significant we could not control for some confounding factors so cannot exclude the possibility that there was confounding by indication or lifestyle. The rate of spontaneous abortion among both exposed and unexposed women was low compared to other studies. This likely reflects the way data is captured in New Zealand (only data on spontaneous abortions managed in hospital are collected). In addition, in this study we excluded ectopic pregnancy and molar pregnancy and these are often included in commonly cited spontaneous abortion rates. In recent years, guidelines have moved toward recommending conservative management as a first line and these women will be largely missing in the NMDS. Studies have found that up to 50% of spontaneous abortions are managed at home, [18] which from our data would estimate the general rate of spontaneous abortion in New Zealand as 12.62%.

AEDs are commonly used to manage other serious psychiatric disorders such as bipolar disorder. Currently there are a paucity of data regarding the association between bipolar disorder and rates of spontaneous abortion. Studies examining the effect of untreated psychiatric illness on obstetrical outcome have found that antenatal stress and anxiety are linked to adverse outcomes such as preterm birth and low birth-weight [19, 20]. It is unknown whether bipolar disorder increases the risk of spontaneous abortion, however it may be possible that symptoms of bipolar disorder or associated behaviours, such as smoking [21], may increase the likelihood of spontaneous abortion and contribute to confounding by indication in this study.

Studies which have included pregnancy termination data have found high rates of termination among women who have taken AEDs [22, 23]. One study found higher pregnancy termination rates among women taking AEDs compared with women not taking AEDs and found the rate of termination was particularly high among women taking AEDs for conditions other than epilepsy [22]. We included pregnancy termination data in this study and found a small but statistically significant decrease in the rate of pregnancy terminations in women who had been dispensed AEDs. There is a possibility that this rate is underestimated due to the termination data missing from private clinics.

International studies looking at AED use among women of child-bearing potential have found that their use is increasing due to increased and wider availability of the newer generation AEDs [11, 24–26]. A study from the UK investigating AED use among adolescent girls found a significant decrease in prescriptions for carbamazepine and sodium valproate and a 10-fold increase in prescriptions for lamotrigine over the same time period [25]. Our results appear to confirm this trend in New Zealand, with overall use of AEDs in women of child-bearing potential increasing over time.

A study in the US [26], found a significant increase in AED prescribing in pregnant women as a result of increasing use of AEDs among pregnant women with diagnoses for psychiatric and pain disorders but not among those with epilepsy diagnoses. In another study, the overall rate of AED use among pregnant women enrolled in Medicaid did not change significantly over time however use of older generation AEDs declined while use of the newer generation AEDs increased [27]. This present study yielded similar results as the number of pregnant women using AEDs in our study did not vary as much over the time period but showed a similar trend towards decreased use of sodium valproate.

### Strengths

The NMDS captures all births, pregnancy terminations and spontaneous abortions which occur in public hospitals within New Zealand. All AEDs in New Zealand are prescription medicines, therefore the Pharmaceutical Collection captures virtually 100% of the dispensing of AEDs in community pharmacies in New Zealand. By combining the Pharmaceutical Collection and the NMDS, data from the majority of the female population in New Zealand who are pregnant or of child-bearing age and exposed to AEDs over a six year period is captured. Deriving this data from the whole population reduces the risk of selection bias and there is also no loss to follow up of the study subjects. In addition it allows the capture of information about AED use among women using them for any condition rather than only for a particular condition such as epilepsy.

### Limitations

Data regarding pregnancy termination in private hospitals and spontaneous abortions not managed in hospital are missing from the NMDS. This accounts for approximately 5000 terminations per year and an unknown number of early spontaneous abortions. The total number of terminations and spontaneous abortions are therefore underestimated by the NMDS. If the pattern of health care utilisation is evenly distributed between exposed and unexposed women then observed differences are valid. The determination of the exposure window in this study was unspecific to some extent and there may be some women not captured by this method. The narrow window increases the likelihood of exposure to AEDs while pregnant in the cohort of women identified.

The NMDS had incomplete information on factors such as smoking status, diagnosis, education of mother, individual patient living circumstances, use of unsubsidized medicines or over-the-counter medicine use. These factors can influence pregnancy outcomes such as the likelihood of pregnancy termination or spontaneous abortion. Without this information it was difficult to control for confounding therefore there is a possibility the differences in outcomes seen between women dispensed AEDs and those not dispensed AEDs are the result of lifestyle factors or the underlying condition AEDs are being used to treat. Using administrative data assumes that the women takes the medicine as prescribed, at the time it is dispensed and that they take it at all. Studies investigating the correlation between self-reported medicine use and administrative data have found a good correlation for chronic conditions such as epilepsy [28].

## Conclusions

This study found the rates of AED use are increasing among women of child-bearing potential in New Zealand with General Practitioners being the most common prescribers of AEDs to this group. This has implications for education to primary care practitioners and women in this age group as it is likely that this trend will continue, seeing more women exposed to AEDs during pregnancy both in New Zealand and internationally. Along with the well documented risk of foetal malformations with some AEDs this study found there may be additional risks for pregnant women using AEDs such as an elevated risk of spontaneous abortion.

## Abbreviations

AED: Antiepileptic drug
NHI: National health index
NMDS: National Minimum dataset (hospital discharges)
